# Comparison of transoral and bilateral axillo-breast approach endoscopic thyroidectomy: multicentre study

**DOI:** 10.1093/bjsopen/zrag077

**Published:** 2026-06-16

**Authors:** Jiann-Ming Wu, Ting-Chun Kuo, Kuen-Yuan Chen, Shang-Ming Tseng, Yi-Jhih Tsai, Ming-Tsan Lin, Chin-Hao Chang, Ming-Hsun Wu

**Affiliations:** Department of Surgery, Far Eastern Memorial Hospital, New Taipei City, Taiwan; Department of Surgery, National Taiwan University Hospital, Taipei, Taiwan; Department of Surgery, National Taiwan University Hospital, Taipei, Taiwan; Department of Surgery, National Taiwan University Cancer Center, Taipei, Taiwan; Department of Surgery, National Taiwan University Hospital, Taipei, Taiwan; Department of Surgery, National Taiwan University Hospital Hsin-Chu Biomedical Park Branch, Hsin-Chu, Taiwan; Department of Surgery, National Taiwan University Hospital, Taipei, Taiwan; Department of Medical Research, National Taiwan University Hospital and National Taiwan University, Taipei, Taiwan; Department of Surgery, National Taiwan University Hospital, Taipei, Taiwan

**Keywords:** remote-access thyroid surgery, propensity score matching, minimally invasive thyroid surgery, surgical outcomes

## Abstract

**Background:**

Remote-access thyroidectomy techniques such as the transoral endoscopic thyroidectomy vestibular approach (TOETVA) and bilateral axillo-breast approach (BABA) offer cosmetic advantages, yet multicentre evidence comparing perioperative safety, operative efficiency, and histopathological integrity is limited.

**Methods:**

This retrospective multicentre cohort study included consecutive adult patients who underwent TOETVA or BABA at four tertiary referral centres between 1 January 2020 and 31 December 2022. Surgeons had surpassed the learning curve. Propensity score matching (1 : 1) was performed based on demographic and clinical variables. Patients with < 12 months follow-up were excluded. Data on operative time (total and phase-specific), postoperative pain, complications, specimen integrity (fragmentation and capsule status), length of hospital stay, reoperations, and mid-term outcome measures, including recurrence, were collected.

**Results:**

For 507 eligible patients (TOETVA, 216; BABA, 291), matching achieved balanced baseline characteristics. TOETVA had longer operative time than BABA (125.6 *versus* 97.7 minutes; *P* < 0.001), with faster flap creation but slower thyroidectomy. Complication rates were similar for TOETVA and BABA, including vocal cord palsy (1.1% *versus* 2.8%, respectively) and hypoparathyroidism (2.2% *versus* 3.3%, respectively). TOETVA had higher rates of specimen disruption (12.8% *versus* 6.1%; *P* = 0.031), whereas tumour capsule integrity was comparable. Bilateral thyroidectomy independently prolonged operative time in both groups; tumour size and male sex also increased operative time in BABA. Mid-term recurrence was uncommon, although recurrence after BABA more often required open or chest wall incisions, whereas recurrence after TOETVA was managed through a limited cervical approach.

**Conclusion:**

TOETVA and BABA are safe and effective remote-access thyroidectomy options, each with distinct technical considerations. TOETVA offers a scarless mid-line corridor with less postoperative discomfort but carries a higher risk of specimen disruption, whereas BABA provides broader exposure and shorter thyroidectomy time but is more affected by anatomical and tumour factors. These findings support evidence-based surgical planning and individualized patient counselling.

## Teaser

In this multicentre propensity score-matched study of 507 patients, transoral endoscopic thyroidectomy (TOETVA) and bilateral axillo-breast approach (BABA) exhibited comparable safety and favourable midterm outcomes. TOETVA was associated with longer operative time but less postoperative pain and a shorter hospital stay, whereas thyroidectomy time was shorter and specimen integrity was more consistent with BABA. These findings highlight key technical trade-offs and support individualized surgical planning.

## Introduction

Thyroid nodules are common and, although most are benign, surgical removal is indicated for suspected malignancy, compressive symptoms, or cosmetic concerns. Open thyroidectomy remains the gold standard for safety and efficacy but leaves a visible cervical scar, prompting the development of remote-access, minimally invasive alternatives.

The transoral endoscopic thyroidectomy vestibular approach (TOETVA) provides a mid-line, scarless route with a shorter working distance to the thyroid, whereas the bilateral axillo-breast approach (BABA) offers wider exposure and preserves conventional operative orientation. However, the narrow extraction tract with TOETVA may compromise specimen integrity, whereas BABA requires more extensive flap dissection and may result in anterior chest discomfort^1–3^. Both approaches demand advanced endoscopic expertise^4,5^.

Despite increasing global adoption, comparative multicentre data evaluating perioperative safety, specimen integrity, and feasibility of reoperation for TOETVA and BABA remain limited^6,7^. Previous studies have largely focused on short-term outcomes or cosmetic satisfaction without addressing mid-term findings or reoperation scenarios^6–8^.

Therefore, the aim of this multicentre study was to use propensity score matching (PSM) to compare TOETVA and BABA in terms of surgical safety, efficiency, and histopathological integrity. In addition, mid-term follow-up outcomes were analysed to provide evidence-based guidance for surgical planning and patient counselling.

## Methods

### Study design and setting

This retrospective multicentre cohort study was conducted at National Taiwan University Hospital (NTUH) and its branches (Biomedical Park Hospital and Cancer Center), as well as at Far Eastern Memorial Hospital (FEMH). All consecutive patients undergoing TOETVA or BABA thyroidectomy between 1 January 2020 and 31 December 2022 were included. Surgeries were performed by surgeons beyond the learning curve (> 2 years experience and ≥ 30 cases of endoscopic thyroidectomy annually).

The institutional review boards of all centres approved the study, and the requirement for informed consent was waived due to the retrospective study design. The study was registered at ClinicalTrials.gov (NCT04569513) and was conducted in accordance with the Declaration of Helsinki.

### Participants

Eligible patients were aged 18–65 years and underwent TOETVA or BABA for benign or malignant thyroid disease. The TOETVA and BABA surgical approaches were explained to all patients before surgery. Patients could choose their preferred surgical approach. Exclusion criteria were incomplete follow-up (< 1 year), severe systemic disease (American Society of Anesthesiologists class IV–V), concurrent major head and neck procedures, or other remote-access approaches.

### Propensity score matching

To reduce selection bias, 1:1 nearest-neighbour PSM without replacement was performed. Propensity scores were generated using logistic regression including body mass index (BMI), tumour size, histological diagnosis, extent of thyroidectomy, thyroiditis, cardiovascular disease, and diabetes. A calliper of 0.1 of the standard deviation (s.d.) of the logit of the propensity score was applied, and patients outside the region of common support were excluded. Matching quality was assessed using standardized mean differences (SMDs), with an SMD ≤ 0.1 indicating acceptable balance.

### Outcome measures

The outcome measures assessed in this study were operating time, postoperative pain, safety and complications, and reoperations and mid-term outcomes.

The total operating room time was defined as the duration of patient presence in the theatre. Operative time was measured from skin incision to closure and subdivided into incision to strap muscle separation (flap creation) and strap muscle separation to closure (thyroidectomy phase).

Postoperative pain was assessed using a ten-point visual analogue scale (VAS). Scores were recorded at 8 hours (h) after surgery for all patients and at 16 h for patients still hospitalized. For intergroup comparisons, VAS scores at 8 h were used as the standardized measure.

Safety and complications were assessed on the basis of vocal cord function and hypoparathyroidism. Vocal cord function was routinely evaluated by ultrasonography^9^, with direct laryngoscopy performed if dysfunction was suspected. Vocal cord palsy or hypoparathyroidism persisting > 12 months was considered permanent^7^.

Reoperations were defined as any secondary thyroid or neck surgery performed after the index procedure. Patients were followed up at 1, 6, and 12 months after surgery and annually thereafter. Mid-term outcomes included tumour recurrence, persistent symptoms, and late complications.

### Statistical analysis

After PSM, paired *t* tests were used to compare continuous variables, and McNemar tests were used to compare paired categorical outcomes at the patient level. Covariate balance before and after PAM was evaluated using SMDs.

To evaluate factors associated with operative time as a continuous variable, Pearson correlation coefficients (*r*) were used for continuous predictors, and point-biserial correlations (*r*) were used for binary predictors. Variables with *P* < 0.20 in univariable analysis or judged clinically relevant were entered into separate multivariable linear regression models for the TOETVA and BABA cohorts. Model performance was evaluated using *R*^2^, and regression parameters were reported as correlations (*r*), coefficients (β), standardized β, the standard error (s.e.), and 95% confidence interval (c.i.).

For non-paired, specimen-level categorical outcomes (for example, specimen disruption, tumour capsule integrity), comparisons were performed using χ^2^ tests, or Fisher’s exact tests when expected cell counts were < 5.

All statistical tests were two-sided, with *P* < 0.05 considered statistically significant. Analyses were performed using SAS^®^ version 9.4 (SAS Institute, Cary, NC, USA).

## Results

**Fig. 1:**
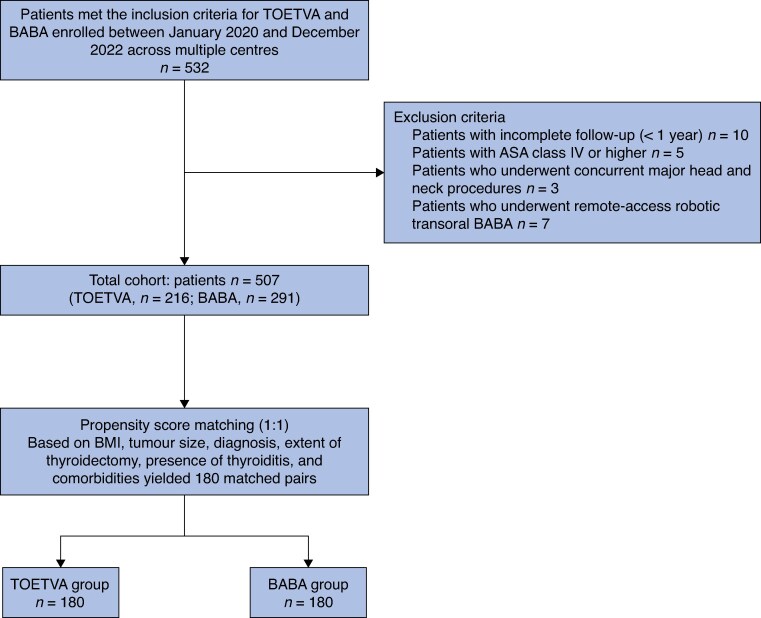
Flow diagram of patient enrolment, exclusion, and propensity score matching TOETVA, transoral endoscopic thyroidectomy vestibular approach; BABA, bilateral axillo-breast approach; ASA, American Society of Anesthesiologists; BMI, body mass index.

Among 3710 thyroidectomies performed during the study period, 532 patients met the inclusion criteria for TOETVA or BABA. After excluding 25 ineligible patients (ten with follow-up < 1 year, five with American Society of Anesthesiologists class IV or higher, three who underwent concurrent major head and neck procedures, and seven who underwent robotic-assisted remote-access thyroidectomy), 507 patients were included in the final analysis (TOETVA, 216; BABA, 291; *Fig. 1*).

Before PSM, patients undergoing TOETVA had a lower BMI, smaller dominant tumours, and fewer bilateral procedures, with fewer patients having thyroiditis, cardiovascular disease, or diabetes (all *P* < 0.05), reflecting approach selection bias. After PSM, no significant demographic or clinical differences were observed between the two groups (*Table 1* and *Fig. S1*).

**Table 1:** Baseline demographic and clinical characteristics before and after propensity score matching

	Before matching				After matching			
	TOETVA (*n* = 216)	BABA (*n* = 291)	P*	SMD†	TOETVA (*n* = 180)	BABA (*n* = 180)	P*	SMD†
Age‡ (years), mean(s.d.)	44.64(11.80)	44.51(10.61)	0.930	0.01	45.02(12.15)	44.03(10.48)	0.408	0.09
**Sex**§			0.132	0.14			0.873	0.02
Male	26 (12.0%)	49 (16.8%)			22 (12.2%)	23 (12.8%)		
Female	190 (88.0%)	242 (83.2%)			158 (87.8%)	157 (87.2%)		
BMI‡ (kg/m^2^), mean(s.d.)	22.90(3.57)	24.70(4.72)	< 0.001	0.43	23.17(3.68)	23.32(3.47)	0.697	0.04
Tumour size‡¶ (cm), mean(s.d.)	2.47(1.77)	2.87(2.04)	0.020	0.21	2.49(1.82)	2.64(1.95)	0.448	0.08
Malignancy§	82 (38.0%)	114 (39.2%)	0.781	0.02	72 (40.0%)	68 (37.8%)	0.665	0.05
Unilateral thyroidectomy§	169 (78.2%)	187 (64.3%)	< 0.001	0.31	134 (74.4%)	136 (75.6%)	0.808	0.03
Thyroiditis§#	0 (0.0%)	19 (6.53%)	< 0.001	0.37	0 (0.0%)	0 (0.0%)	NA	NA
Cardiovascular disease§#	0 (0.0%)	11 (3.8%)	0.004	0.28	0 (0.0%)	0 (0.0%)	NA	NA
Diabetes§#	1 (0.5%)	18 (6.2%)	< 0.001	0.32	1 (0.6%)	1 (0.6%)	1.000	0.00

Values are *n* (%) unless otherwise stated. †SMDs were used to assess covariate balance, with SMD ≤ 0.10 indicating adequate balance after matching. ¶Tumour size refers to maximum tumour diameter. TOETVA, transoral endoscopic thyroidectomy vestibular approach; BABA, bilateral axillo-breast approach; SMD, standardized mean difference; s.d., standard deviation; BMI, body mass index; NA, not applicable. *Calculated using two-sided tests with significance set at *P* < 0.05. ‡Compared using independent-samples *t* test. §Compared using χ^2^ test or Fisher exact test when appropriate. #Fisher’s exact test was used when expected cell counts were < 5.

TOETVA was associated with a longer operative time (mean(s.d.) 125.6(37.9) *versus* 97.7(29.7) minutes (min); mean difference, 27.9 min; *P* < 0.001). The flap creation phase was faster with TOETVA than BABA (mean(s.d.) 16.7(5.6) *versus* 22.9(7.5) min; mean difference, −6.2 min; *P* < 0.001), whereas the thyroidectomy phase was shorter with BABA (mean(s.d.) 74.8(30.2) *versus* 108.9(37.2) min; mean difference, 34.1 min; *P* < 0.001). Thyroidectomy-related complication rates were comparable between the TOETVA and BABA groups (3.3% *versus* 6.1%, respectively; risk difference, −2.8%; *P* = 0.302), including vocal cord palsy (1.1% *versus* 2.8%, respectively; risk difference, −1.7%; *P* = 0.450) and hypoparathyroidism (2.2% *versus* 3.3%, respectively; risk difference, −1.1%; *P* = 0.752), with all vocal cord dysfunction confirmed by postoperative laryngoscopy. Procedure-specific morbidity differed: lower chin numbness occurred exclusively after TOETVA (6.1%), whereas anterior chest discomfort was unique to BABA (9.4%; risk difference, −3.3%; *P* = 0.307), although neither difference reached statistical significance.

Conversion to open surgery was rare with both TOETVA and BABA (0.0% *versus* 2.2%, respectively; risk difference, −2.2%; *P* = 0.134), and no perioperative infection or mortality occurred. After TOETVA, postoperative pain was lower (mean(s.d.) VAS 1.1(0.3) *versus* 1.4(0.9); mean difference, −0.3; *P* < 0.001) and the postoperative length of hospital stay was shorter (mean(s.d.) 1.1(0.4) *versus* 2.0(0.5) days; mean difference, −0.8 days; *P* < 0.001). The length of follow-up was comparable between the TOETVA and BABA groups (median 34 (interquartile range (i.q.r.) 29–41) *versus* 36 (i.q.r. 29–42) months, respectively; *Table 2*).

**Table 2:** Intraoperative parameters and surgical outcomes after matching in TOETVA *versus* BABA

	TOETVA (*n* = 180)	BABA (*n* = 180)	Mean/risk difference*	*P*†
**Operative time‡ (min), mean(s.d.)**	125.60(37.90)	97.66(29.72)	27.94 (20.68, 35.20)	< 0.001
Flap creation‡ (min), mean(s.d.)	16.72(5.56)	22.87(7.50)	−6.15 (−7.47, −4.83)	< 0.001
Thyroidectomy‡ (min), mean(s.d.)	108.88(37.19)	74.79(30.20)	34.09 (26.85, 41.33)	< 0.001
**Thyroidectomy-related complications§**	6 (3.3%)	11 (6.1%)	−2.78 (−6.98, 1.42)	0.302
Vocal cord palsy§	2 (1.1%)	5 (2.8%)	−1.67 (−4.54, 1.20)	0.450
Transient	2 (1.1%)	4 (2.2%)	−1.11 (−3.77, 1.55)	0.683
Permanent	0 (0.0%)	1 (0.6%)	−0.56 (−1.64, 0.53)	0.999
Hypoparathyroidism§	4 (2.2%)	6 (3.3%)	−1.11 (−4.55, 2.33)	0.752
Transient	3 (1.7%)	4 (2.2%)	−0.56 (−3.44, 2.32)	0.999
Permanent	1 (0.6%)	2 (1.1%)	−0.56 (−2.44, 1.33)	0.999
**Procedure-specific complications§**	11 (6.1%)	17 (9.4%)	−3.33 (−8.65, 1.98)	0.310
Lower chin numbness	11 (6.1%)	0 (0.0%)	6.11 (2.61, 9.61)	0.003
Anterior chest wall numbness	0 (0.0%)	17 (9.4%)	−9.44 (−13.72, −5.17)	< 0.001
Conversion to open surgery¶	0 (0.0%)	4 (2.2%)	−2.22 (−5.01, 0.56)	0.134
Infection	0 (0.0%)	0 (0.0%)	NA	NA
VAS pain scores at 8 h‡, mean(s.d.)	1.11(0.32)	1.43(0.9)	−0.32 (−0.45, −0.20)	< 0.001
Postoperative stay‡ (days), mean(s.d.)	1.11(0.39)	1.96(0.50)	−0.84 (−0.75, −0.43)	< 0.001
Follow-up duration¶ (months), median (i.q.r.)	34 (29–41)	36 (29–42)	−1.00 (−3.00, 1.50)	0.184

Values are *n* (%) unless otherwise stated. *Values in parentheses are 95% confidence intervals. Mean differences and 95% confidence intervals were calculated using paired *t* tests; risk differences and 95% confidence intervals were calculated for categorical outcomes. TOETVA, transoral endoscopic thyroidectomy vestibular approach; BABA, bilateral axillo-breast approach; min, minutes; s.d., standard deviation; NA, not applicable; VAS, visual analogue scale; h, hours; i.q.r., interquartile range. †Two-sided *P* < 0.05 was considered statistically significant. ‡Compared using paired *t* tests. §Compared using the McNemar test for paired binary outcomes. For outcomes measured at the organ level, a χ^2^ test or Fisher exact test was used as appropriate. ¶Follow-up duration was calculated from the date of initial surgery to the most recent clinic visit or reoperation. Median differences and 95% confidence intervals were estimated using non-parametric bootstrap methods, and between-group comparisons of median follow-up duration were performed using the Wilcoxon signed-rank test.

Adenomatous goitre was the most common benign diagnosis in both groups. Well-differentiated tumours of uncertain malignant potential (WDT-UMP) were more frequent in the TOETVA than BABA group (4.4% *versus* 1.7%; mean difference, 2.8%; *P* = 0.221; *Table 3*), although the difference was not statistically significant. Specimen disruption occurred more often in the TOETVA than BABA group (12.8% *versus* 6.1%; mean difference, 6.7%; *P* = 0.031), whereas incomplete tumour capsule integrity was comparable between the two groups (3.3% *versus* 2.2%, respectively; mean difference, 1.1%; *P* = 0.748; *Table 3*). Among follicular neoplasms, incomplete capsule integrity was observed in 7.3% and 3.8% of patients in the TOETVA and BABA groups (mean difference, 3.6%; *P* = 0.650; *Table 3*).

**Table 3:** Histopathological findings and specimen integrity in TOETVA and BABA

	TOETVA (*n* = 180)	BABA (*n* = 180)	Risk difference*	*P*†
**Histopathological finding**				
Adenomatous goitre	71 (39.4%)	66 (36.7%)	2.80 (−7.20, 12.80)	0.664
PTC	68 (37.8%)	61 (33.9%)	3.90 (−6.00, 13.80)	0.510
Follicular/Hürthle cell adenoma	27 (15.0%)	36 (20.0%)	−5.00 (−12.80, 2.80)	0.212
NIFTP	3 (1.7%)	11 (6.1%)	−4.40 (−8.40, −0.50)†	0.029
FTC	3 (1.7%)	3 (1.7%)	0.00 (−2.60, 2.60)†	> 0.999
WDT-UMP	8 (4.4%)	3 (1.7%)	2.78 (−0.80, 6.30)†	0.221
**Specimen integrity**				
Specimen disrupted	23 (12.8%)	11 (6.1%)	6.67 (−0.70, 12.70)	0.031
Incomplete tumour capsule integrity	6 (3.3%)	4 (2.2%)	1.10 (−2.30, 4.50)	0.748
FN with incomplete tumour capsule integrity§	3 of 41 (7.3%)	2 of 53 (3.8%)	3.60 (−5.90, 13.00)†	0.650

Values are *n* (%) unless otherwise stated. *Values in parentheses are 95% confidence intervals. Risk differences and 95% confidence intervals were calculated using Wald or Newcombe hybrid score methods, depending on event frequency. Comparisons between matched groups were performed using χ^2^ or Fisher exact tests as appropriate. §FN includes follicular/Hürthle cell adenoma, NIFTP, FTC, and WDT-UMP; data show the number of FNs with incomplete tumour capsule integrity of the total number of FNs, with percentages in parentheses. TOETVA, transoral endoscopic thyroidectomy vestibular approach; BABA, bilateral axillo-breast approach; PTC, papillary thyroid cancer; NIFTP, non-invasive follicular thyroid neoplasm with papillary-like nuclear features; FTC, follicular thyroid carcinoma; WDT-UMP, well-differentiated tumour of uncertain malignant potential; FN, follicular neoplasm. †*P* < 0.05 was considered statistically significant.

On univariable analysis, only bilateral thyroidectomy was associated with prolonged operative time in the TOETVA group (*r* = 0.198; *P* = 0.008; *Table 4*). In multivariable linear regression analysis, bilateral thyroidectomy remained the sole independent predictor of operative time (β = 17.20 min; 95% c.i., 4.59 to 29.81; *P* = 0.008; *R*^2^ = 0.034). In contrast, in the BABA group, male sex (*r* = 0.178; *P* = 0.017), tumour size (*r* = 0.173; *P* = 0.020), and bilateral thyroidectomy (*r* = 0.178; *P* = 0.017) were associated with operative time in univariable analysis, and all remained independent predictors in multivariable analysis (male sex: β = 19.10 min (95% c.i. 6.48 to 31.70; *P* = 0.001); tumour size: β = 2.99 min/cm (95% c.i. 0.84 to 5.14; *P* = 0.007); bilateral thyroidectomy: β = 16.60 min (95% c.i. 6.72 to 26.50; *P* = 0.001); model *R*^2^ = 0.100). These findings suggest that operative time in BABA is more susceptible to patient- and tumour-related factors, whereas TOETVA appears less affected by these variables (*Table 4*).

**Table 4:** Univariable and multivariable linear regression analysis of factors associated with operation time after matching

	Univariable analysis		Multivariable linear regression*				
	Correlation (*r*)	*P*	Coefficient (β)	Standardized β	s.e.	95% c.i.	*P*
**TOETVA (*n* = 180)**							
Age	0.003	0.969					
Male sex	−0.023	0.759					
BMI	−0.030	0.691					
Tumour size	0.036	0.632					
Malignancy	0.115	0.124					
Bilateral thyroidectomy	0.198	0.008	17.20	0.20	6.37	(4.59, 29.81)	0.008
**BABA (*n*** **=** **180)**							
Age	−0.035	0.637					
Male sex	0.178	0.017	19.10	0.22	6.40	(6.48, 31.70)	0.003
BMI	0.114	0.129					
Tumour size	0.173	0.020	2.99	0.20	1.09	(0.84, 5.14)	0.007
Malignancy	−0.087	0.244					
Bilateral thyroidectomy	0.178	0.017	16.60	0.24	5.01	(6.72, 26.50)	0.001

Values in parentheses are 95% confidence intervals. Univariable correlations were calculated using Pearson correlation for continuous predictors (age, BMI, tumour size) and point-biserial correlation for binary variables (sex, malignancy, bilateral thyroidectomy). s.e., standard error; TOETVA, transoral endoscopic thyroidectomy vestibular approach; BMI, body mass index; BABA, bilateral axillo-breast approach. *Variables with *P* < 0.20 in the univariable analysis or of clinical relevance were included in multivariable linear regression models. Multivariable regression reports unstandardized coefficients (β), standardized β values, the s.e., and 95% confidence intervals. Model fit was assessed with *R*^2^ (TOETVA *R*^2^ = 0.034; BABA *R*^2^ = 0.100). All tests were two-sided, with *P* < 0.05 considered statistically significant.

**Fig. 2:**
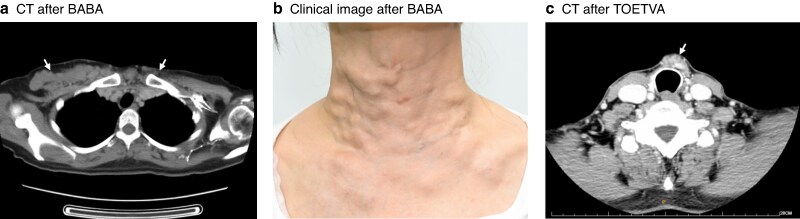
Representative images showing recurrence and tumour seeding after BABA and TOETVA requiring reoperation **a, b** Patient after BABA. The initial thyroidectomy pathology was follicular adenoma, with reoperation revealing follicular thyroid carcinoma. **a** Contrast-enhanced CT showing multiple recurrent lesions (arrows) in the anterior neck and chest within the previously dissected flap. **b** Clinical photograph showing multiple recurrences on the patient’s neck and anterior chest, necessitating wide dissection and additional skin incisions. **c** Patient after TOETVA. The initial pathology was follicular adenoma, and reoperative pathology confirmed follicular thyroid carcinoma. Contrast-enhanced CT demonstrated a suspicious recurrent lesion (arrow) in the suprastrap muscle area of the anterior neck within the previously dissected flap. BABA, bilateral axillo-breast approach; TOETVA, transoral endoscopic thyroidectomy vestibular approach; CT, computed tomography.

In the total cohort (507 patients), completion thyroidectomy was required in a subset of patients. Four TOETVA patients underwent re-TOETVA, with one conversion to open surgery, whereas seven BABA patients underwent re-BABA, of whom five required open conversion. Subcutaneous tumour seeding or locoregional recurrence occurred in one TOETVA patient and two BABA patients, all managed by surgical resection. Recurrence in the TOETVA group was treated through a conventional cervical incision, whereas recurrence in the BABA group involved diffuse or distant chest wall seeding, necessitating additional anterior chest incisions for wide excision (*Fig. 2*).

## Discussion

In this multicentre propensity-matched analysis, both TOETVA and BABA were safe, feasible, and highly reproducible approaches for thyroidectomy in experienced hands. TOETVA offered shorter postoperative recovery, lower pain scores, and faster working space creation, whereas BABA provided shorter total operative time and more reliable specimen integrity. Although rare events such as tract seeding or reoperation-related challenges have been reported^7,10–12^, appropriate patient selection and meticulous operative technique enable effective prevention and management of such complications or recurrences. The findings of this study provide evidence-based guidance for surgical planning, approach selection, and patient counselling in minimally invasive thyroid surgery.

This study demonstrated that TOETVA was preferentially selected for patients with a lower BMI, smaller tumours, unilateral disease, and fewer co-morbidities, including thyroiditis, highlighting baseline differences and inherent selection bias in the choice of surgical approach. To address these imbalances, 1 : 1 PSM was performed, effectively minimizing preoperative differences and enabling a balanced comparison of perioperative safety, operative efficiency, and specimen integrity between TOETVA and BABA.

Postoperative cervical scarring remains a major cosmetic concern, strongly influencing patient preference and driving the development of remote-access approaches^13–15^. Since its introduction by Anuwong *et al*.^16–18^, TOETVA has provided a truly scarless mid-line route with a shorter working distance compared with other remote-access techniques, including BABA^19^. Both approaches offer superior cosmetic outcomes and quality of life compared with conventional open thyroidectomy, with TOETVA yielding the most favourable scarless results^20^. These cosmetic advantages continue to motivate the adoption of remote-access techniques worldwide, particularly in younger and cosmetically motivated patient populations.

In the present matched analysis, overall complication rates were low and comparable between approaches, with the incidence of vocal cord palsy and hypoparathyroidism within or below previously reported ranges (transient recurrent laryngeal nerve injury 1–10%, permanent < 2%)^21–25^. This aligns with contemporary series demonstrating that remote-access thyroidectomy can achieve safety outcomes equivalent to open surgery when performed by experienced teams^7,16^. Other complications, such as bleeding, seroma, and infection, were rare, supporting the safety and reproducibility of both approaches in experienced hands^26^. Although overall morbidity was similar, procedure-specific sensory changes differed: chin numbness occurred only after TOETVA, whereas anterior chest discomfort occurred only after BABA. These differences reflect the distinct anatomical dissection planes required for each approach and should be incorporated into preoperative counselling.

Operative efficiency differed between techniques. BABA was associated with shorter thyroidectomy time, likely owing to its wider working space and four-port configuration (including the endoscopic port), which facilitates ergonomic triangulation and reduces instrument collisions compared with the three-port TOETVA setup. Flap creation was faster in TOETVA due to minimal dissection requirements.

Specimen integrity emerged as a key differentiator. TOETVA showed higher rates of specimen fragmentation and capsular disruption, a finding consistent with previous reports of transoral extraction challenges^7,8^. Intact capsule and vascular architecture are critical for accurate histopathological assessment, particularly in follicular-patterned lesions where capsular or vascular invasion determines the diagnosis of carcinoma^27–30^.

Importantly, in the present study, the diagnosis of WDT-UMP was limited to specimens with preserved and evaluable tumour capsules, and lesions with insufficient capsule integrity were not classified as WDT-UMP. Although differences in capsule integrity were not statistically significant, the observed trend towards higher disruption with TOETVA underscores the technical vulnerability inherent to transoral retrieval and reinforces the need for meticulous extraction techniques and cautious patient selection^31,32^. Although minor specimen disruption was more frequent in TOETVA, it did not appear to compromise margin assessment, tumour staging, or the accuracy of histopathological classification in the present cohort. Nevertheless, such disruption has potential clinical relevance, particularly in follicular-patterned neoplasms, and should be minimized to ensure reliable oncological evaluation.

Rare cases of tract seeding or surgical bed recurrence underscore the importance of maintaining capsular integrity and ensuring complete, en bloc tissue retrieval^33–38^. Reoperation for such recurrences can be more challenging after BABA due to wider dissection and longer operative tracts, whereas recurrences following TOETVA are typically localized and manageable through limited cervical incisions^39–41^. For patients requiring completion thyroidectomy on the contralateral side, endoscopic reoperation may be safely performed in select patients; however, open surgery remains the standard for extensive or recurrent disease^42^.

Operative time in endoscopic thyroidectomy, as demonstrated in the present study, is influenced by procedure type, tumour characteristics, the extent of dissection, patient anatomy, and surgical experience^7,43–45^. In the present study, bilateral thyroidectomy was the strongest predictor of prolonged operative duration for both approaches. In addition, male sex and larger tumour size independently prolonged BABA operative time, whereas TOETVA operative time appeared less affected by patient or tumour factors. These findings suggest that operative duration in BABA is particularly sensitive to anatomical and technical challenges, whereas TOETVA exhibits greater procedural uniformity^46,47^.

This study has several limitations. First, its retrospective design introduces inherent risks of unmeasured confounding and selection bias, although PSM mitigated baseline differences. Second, the cohort included only patients treated at high-volume centres by experienced surgeons, which may limit generalizability to lower-volume settings or surgeons early in their learning curve. Third, the maximum follow-up duration of 4 years may be insufficient to assess late oncological recurrences or long-term functional outcomes, particularly in low-risk differentiated thyroid cancer. Fourth, rare adverse events, such as tract seeding, wound-tract recurrence, or distant chest-wall implantation, occurred too infrequently to enable meaningful statistical comparison between TOETVA and BABA. Fifth, granular patient-reported outcomes beyond cosmetic satisfaction, including voice quality, swallowing function, and postoperative sensory symptoms, were not systematically captured. Finally, although specimen integrity was evaluated by dedicated endocrine pathologists, subtle alterations related to endoscopic traction or thermal manipulation may have been underrecognized, especially in minimally invasive approaches where tissue handling is more constrained.

Further prospective multicentre studies with long-term follow-up are warranted to confirm the oncological adequacy, durability, and optimal patient selection for each approach.

## Supplementary Material

zrag077_Supplementary_Data

## Data Availability

The data sets generated and analysed during the present study are not publicly available due to patient confidentiality and institutional policies. However, deidentified data supporting the findings of this study are available from the corresponding author upon reasonable request and with appropriate institutional approvals.
