# Supplementary material for: Comparison of transoral and bilateral axillo-breast approach endoscopic thyroidectomy: multicentre study
Source: BJS Open. 2026 Jun 16;10(3):zrag077. doi: 10.1093/bjsopen/zrag077 (PMC13270485; doi:10.1093/bjsopen/zrag077)
Supplement: zrag077_Supplementary_Data [file zrag077_supplementary_data.docx]

**Comparison of Transoral and BABA Endoscopic Thyroidectomy: Multicenter Study**

Jiann-Ming Wu^1^, MD; Ting-Chun Kuo^2^, MD, PhD, FACS; Kuen-Yuan Chen^2,3^, MD, PhD; Shang-Ming Tseng^2^, MD; Yi-Jhij Tsai^4^, MD; Ming-Tsan Lin^2^, MD, PhD; Chin-Hao Chang^5^, PhD; Ming-Hsun Wu^2^, MD, PhD

^1^Department of Surgery, Far Eastern Memorial Hospital, New Taipei City, Taiwan

^2^Department of Surgery, National Taiwan University Hospital, Taipei, Taiwan

^3^Department of Surgery, National Taiwan University Cancer Center, Taipei, Taiwan

^4^Department of Surgery, National Taiwan University Hospital Hsin-Chu Biomedical Park Branch, Hsin-Chu, Taiwan

^5^Department of Medical Research, National Taiwan University Hospital & National Taiwan University, Taipei, Taiwan

J.-M. Wu and T.-C. Kuo had equal contributions.

**Corresponding author.** Ming-Hsun Wu, MD, PhD, FACS; No.7, Chung Shan S. Rd., Taipei 10002, Taiwan; https://orcid.org/0000-0002-8811-9203.

**Supplementary Materials – Index**

**Supplementary Figures and Tables**

| **eFigure. Covariate Balance Before and After Propensity Score Matching**  Standardized mean differences (SMDs) are shown for each baseline covariate before (blue) and after (red) matching. The vertical dashed lines indicate an absolute SMD of 0.1, representing acceptable covariate balance. Matching achieved substantial reduction in imbalance across all variables.  Abbreviations: DM, diabetes mellitus; CVD, cardiovascular disease; BMI, body mass index. | *page 2* |
| --- | --- |

**Supplementary Figures and Tables**

**
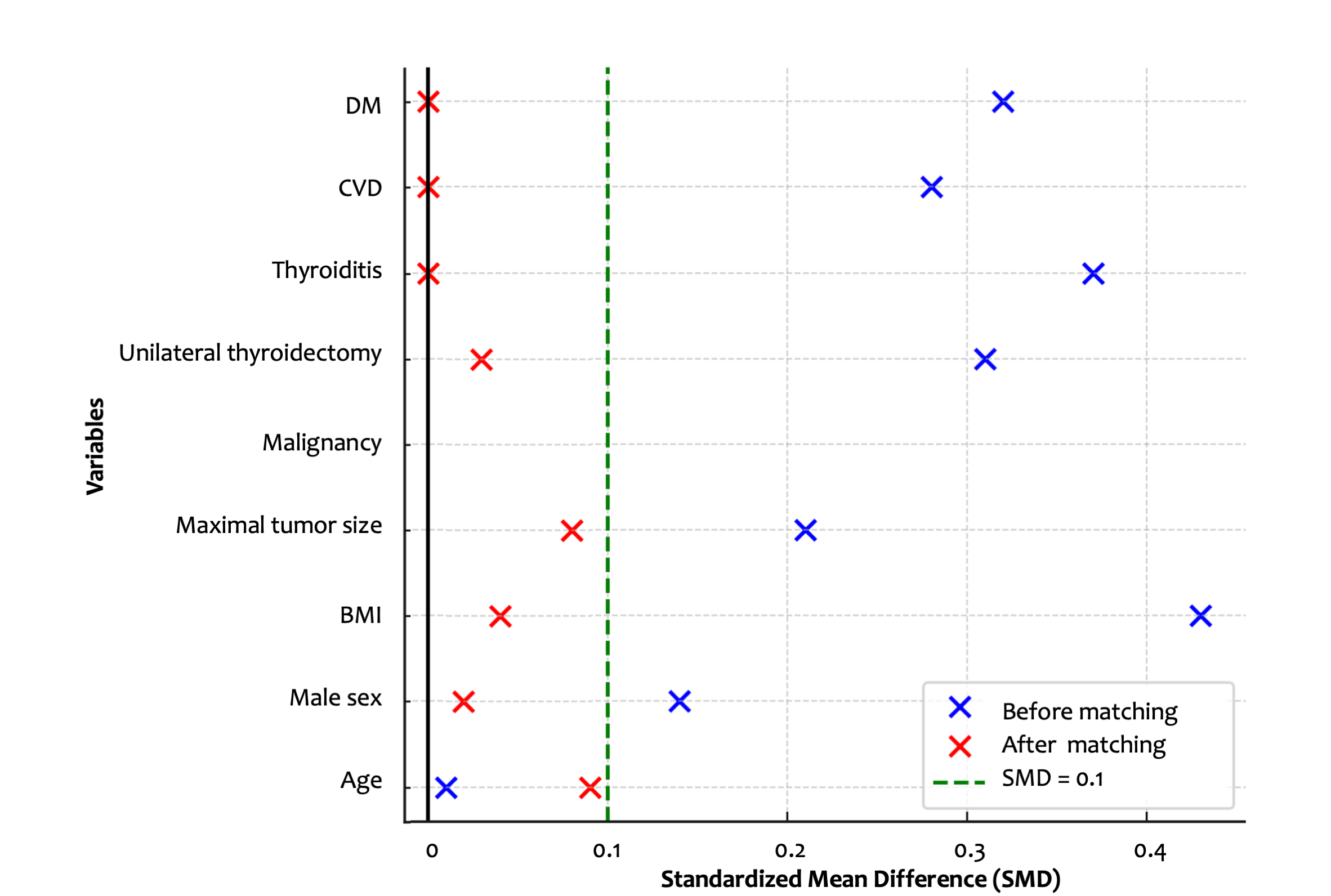
**
